# The Great War: VIII. Sanitation

**Published:** 1918-05-25

**Authors:** 


### THE GREAT WAR.

### VIII.—SANITATION *(concluded)*.

### An Ideal Organisation.

Sanitary inspection and supervision at the Front is carried out on lines of extreme decentralisation. Inspectors on their daily rounds report themselves to the senior officer present, and after their inspection settle the remedy of defects on the spot; only matters which are beyond local action are taken further, then the Sanitary Officer himself confers with the officer commanding the unit concerned, and afterwards, if necessary, with the A.D.M.S. of the division, who either settles the matter himself or refers it to the Divisional Staff. It is a system which works smoothly, pleasantly, and expeditiously, and eliminates correspondence.

### The Workshop.

The workshop is one of the most recent innovations of the war, and one of the most successful. It aims at the supply and upkeep of standardised latrine-seats, urinals, incinerators, meat-safes, notice-boards, and other sanitary appliances for the Army at the Front. At the beginning of the war these appliances had not become fashionable, shallow trench-latrines were official, and burnable rubbish was buried. As, however, the war progressed and remained stationary, deep latrines in dry soil and buckets in wet were substituted, for which fly-proof seats were required. Burning of rubbish also became general, which required incinerators.

The suppression of flies advanced slowly; they abounded in summer, and so food-safes were required. There is no fixed establishment for a workshop; ordinarily it consists of a permanent staff of two bricklayers, three carpenters, one tinsmith, one blacksmith, one sign-painter, supplemented by skilled mechanics from regimental pioneers.

The authorised box of tools is supplemented by loans from the Royal Engineer workshop; the pioneers bring their tools with them.

Wood and other material is supplied by the Royal Engineers, supplemented largely from salvage dumps, by used ration-boxes, and by empty petrol, kerosine, and other tins.

All sanitary requirements are referred to the Sanitary Section, a system which prevents overlapping, effects an incalculable saving of wood and other material, and ensures approved designs.

### Water Supply.

The water supply is controlled by the Sanitary Section; each well is numbered, registered, and placarded with the number of scoops of chloride of lime per water-cart required for sterilisation. Besides wells there are numbers of what are termed "water points" throughout the area; they consist of collections of capacious elevated tanks, into which water is pumped, through iron piping, from various sources, such as artesian borings through impervious strata to great depths into the deep basins, and from selected rivers, wells, or reservoirs, often some miles distant. Every water-cart is regularly inspected, and this inspection is controlled by surprise visits from the Army Mobile Hygiene Laboratory experts, who collect samples of water and test them for bacterial content and chlorine index.

### Disposal of Nightsoil.

There are no sewers or system of drainage in the area of operations in Northern France. Middens are in general use—these in the large towns are cemented and of considerable capacity, and in the villages they are simply pits in the back yard, covered by an ordinary w.c. seat; in both the contents are removed periodically and applied to the land.

The capacity of this accommodation is limited to the requirements of the civil population, so that the needs of our Army require to be met in some other way. The shallow-trench system was a total failure, the ground available for it was insufficient, in dry weather the men would not cover the excreta with dry earth, and in wet weather they could not; in wet weather, also, the sides of the trenches subsided, and a noxious quagmire resulted. Then the nitrifying germs which exist in the superficial layers of some soils failed in this country, and buried excreta remained unchanged for months.

The breakdown of the cherished shallow trench was followed by a long experimental period, during which were tried buckets with removal of contents to deep pits, buckets and incineration, small pans which were emptied by the users into a perpetual fire, and deep trenches of varying sizes and depths. It is only quite recently that the question has reached apparent finality, and the reorganisation of the Sanitary Sections is deeply concerned in the result.

### Group Latrines.

The whole Front is now divided into billeting areas under the care of responsible officers, and each area is divided for conservancy requirements into groups, a central latrine being provided for each.

Wherever possible a trench twelve feet deep, covered by the requisite number of fly-proof seats, and with protection from observation and weather, is provided.

This is the simplest and most satisfactory system from every point of view—a minimum of labour is required, and there are no flies, no unpleasantness, and no danger to health. It is extremely economical and disinfectants are unnecessary. The question of pollution of the sources of drinking-water is not a grave one; it is recognised that the subsoil within range of habita-

☛ Previous articles appeared on Feb. 9 and 23, March 9 and 23, April 6 and 20, and May 4, p. 93.

THE GREAT WAR—(*continued*).

tions is saturated with night soil, and that, consequently, all shallow wells are polluted, and that precautions must be taken accordingly; that there is direct communication between well and privy is frequently unwittingly demonstrated by enthusiastic sanitary parties who disinfect the privy by a generous application of cresol, which soon percolates into the family well with results which impose a severe strain on the "Entente"! Group latrines in towns and in sites having a high ground water-level are provided with buckets, the soil being burnt in Horsfall, or locally constructed brick destructors.

In the trenches enemy observation and aeroplane photography impose reservations on the disposal of excreta; latrine seats and incinerators draw fire, so a wooden cover with a flap door replaces the seat, and burial in an adjacent shell-hole, the incinerator.

PUBLIC URINALS.

Associated with the group latrines is a system of public urinals, in the construction of which empty tins of every description are utilised; they are drained into extensive hermetically sealed caverns. The Sanitary Sections are responsible for their construction and maintenance, which compare favourably with those of the most modern metropolitan types.

INFECTIOUS DISEASES.

On the occurrence of a case of infectious disease in an area, either in military or civilian dwellings, the Sanitary Section officer is informed, and the infected quarter is placarded "Infectious Disease. Out of bounds for troops." Military quarters are evacuated and disinfected, and in the case of civilian dwellings the occupants are instructed and assisted in carrying out the necessary measures of protection, including the inoculation or vaccination of contacts. Sulphur dioxide gas compressed in iron cylinders was mainly used in the disinfection of dwellings in the earlier period of the war, but recently formalin vapour has taken its place.

THE FODEN THRESH DISINFECTOR.

During the first half of the war the official disinfector in the field was an ordinary Thresh, a steam apparatus mounted on a horse-drawn vehicle; this was not efficient, its capacity was limited, and a long exposure was required to produce doubtful results. An improved apparatus was then gradually introduced; which, compared with the old pattern, was as a tractor-drawn howitzer to a horse-drawn field-gun. It consists of twin cylinders of increased capacity, mounted on a steam Foden lorry, the steam from the engine serving the double purpose of traction and disinfection. The steam in the disinfecting chamber being under pressure, the duration of treatment was much shortened, each cycle of loading, steaming, drying, and unloading requiring only one hour.

The capacity is sufficient for the disinfection of seventy blankets, or equivalent underclothing, at a time. One of these machines is authorised for each division and one for each corps' troops. This scale more than meets the requirements in connection with infectious diseases, but is not sufficient to grapple with the destruction of lice, which is further provided for by the Clayton sulphur-vapour installation and by improvised steam disinfectors of the Serbian barrel type.

(*To be continued.*)

